# High inappropriate red blood cell transfusion rate despite low overall use: a real-world multicenter study in 43 Spanish hospitals

**DOI:** 10.3389/fmed.2026.1803092

**Published:** 2026-05-14

**Authors:** Álvaro Tamayo-Velasco, J. V. Llau, Maria J. Colomina, Olga de la Varga-Martínez, Rosalia Navarro-Pérez, Rocío López-Herrero, Jorge Almoguera-Fernández, Marta Alonso-Fernández, María Artiaga-Candia, Ángel Becerra-Bolaños, Natalia Bugueño, Alma Casasempere-Sanus, Laura Contreras-López, María Heredia-Rodríguez, I. de la Fuente-Graciani, Marta de la Rosa-Estadella, Iria de la Torre-Riveiro, Elena del Val-Peciña, Carla Delgado-Martí, Laura Edo-Cebollada, Ricardo Fernández-Fernández, Jorge Fernández-Rodríguez, Carolina Ferrer-Gómez, Marta Giné-Servén, Almudena González-Pereira, A. Guereca-Gala, Aurora Herrera-Soto, M. E. Infantes-Morales, J. M. Marcos-Vidal, Gustavo Illodo-Miramontes, Adrián Martínez-López, Alberto Martínez-Ruiz, Beatriz Martínez-Rafael, Adrián Matute-González, Laura Pariente-Juste, Sonia Pérez-González, Estefania Gómez-Pesquera, A Renedo-Fernández, A. A. Rodríguez-Álvarez, I. Sánchez-Rodríguez, María del Aranzazú Rodríguez-Conesa, Cristina Sánchez-González, Cristina Tobar-Gonzalo, Lorena Varela-Rodríguez, R. Veloso-de Sousa, M. F. Muñoz-Moreno, J. I. García-Sánchez, Felipe Rocha-García, Pablo González-Montes, Patricia Cid-Boo, Gabriel Escudero-Gómez, Nekane Romero-García, Ana Cots, M. J. Peñarrubia-Ponce, Roberto Hornero, Eduardo Santamaría-Vázquez, Rodrigo Poves-Álvarez, Eduardo Tamayo, Marina Varela-Duran, Rafael Badenes, DELPO Study Group

**Affiliations:** 1 Department of Hematology, Clinic University Hospital of Valladolid, Spain; 2 BioCritic, Group for Biomedical Research in Critical care Medicine, Valladolid, Spain; 3 Centro de Investigación Biomédica en Red de Enfermedades Infecciosas (CIBERINFEC), Instituto de Salud Carlos III, Madrid, Spain; 4 Department of Medicine, University of Valladolid, Valladolid, Spain; 5 Biomedical Health Research Institute of Valladolid (IBioVALL), Valladolid, Spain; 6 Department of Anesthesiology and Post-surgical Critical Care, University Hospital Doctor Peset, Valencia, Spain; 7 Department of Surgery, School of Medicine, University of Valencia, Valencia, Spain; 8 Department of Anesthesia, Critical Care and Pain Clinic-Hospital Universitari de Bellvitge-Barcelona-HUB, L’Hospitalet de Llobregat, Spain; 9 Department of Surgery, School of Medicine, Universitat de Barcelona-UB, Barcelona, Spain; 10 Department of Anesthesiology, University Hospital Infanta Leonor, Madrid, Spain; 11 Department of Anesthesiology, Clinic University Hospital San Carlos, Madrid, Spain; 12 Department of Anesthesiology, Clinic University Hospital of Valladolid, Valladolid, Spain; 13 Department of Surgery, University of Valladolid, Valladolid, Spain; 14 Department of Anesthesiology, University Hospital of Fuenlabrada, Madrid, Spain; 15 Department of Anesthesiology, University Hospital Marques de Valdecilla, Santander, Spain; 16 Department of Anesthesiology, University Hospital Ribera Povisa, Vigo, Spain; 17 Department of Anesthesiology, University Hospital of Gran Canaria Doctor Negrin, Las Palmas de Gran Canaria, Spain; 18 Department of Anesthesiology, Clinic Hospital University Sant Joan d’Alicant, Alicante, Spain; 19 Department of Anesthesiology, University Hospital of Manises, Valencia, Spain; 20 Department of Clinical Science, School of Medicine, Universitat de Barcelona-UB, Barcelona, Spain; 21 Health Group, Neuroscience Program, Perioperative Physiopathology and Pain, Institut d’Investigació Biomèdica de Bellvitge- IDIBELL, UB, L’Hospitalet de Llobregat, Spain; 22 Department of Anesthesiology, University Hospital of Salamanca, Salamanca, Spain; 23 Department of Anesthesiology, Hospital Consorci Corporació Sanitària Parc Taulí de Sabadell, Barcelona, Spain; 24 Department of Anesthesiology, University Hospital Álvaro Cunqueiro, Vigo, Spain; 25 Department of Anesthesiology, University Hospital of Donostia, San Sebastian, Spain; 26 Department of Anesthesiology, General Hospital of Castellon, Castellon, Spain; 27 Department of Anesthesiology, University Hospital Lucus Augusti, Lugo, Spain; 28 Department of Anesthesiology, University Hospital of Santiago de Compostela, Santiago de Compostela, Spain; 29 Department of Surgery, Faculty of Medicine, University of Santiago de Compostela, Santiago de Compostela, Spain; 30 Department of Anesthesiology, Valencia General Hospital Consortium, Valencia, Spain; 31 Department of Anesthesiology, Hospital de Sant Pau, Barcelona, Spain; 32 Department of Anesthesiology, University Hospital of A Coruña, A Coruña, Spain; 33 Department of Anesthesiology, University Hospital of Cruces, Bilbao, Spain; 34 Department of Anesthesiology, University Hospital of Cabueñes, Gijón, Spain; 35 Department of Anesthesiology, University Hospital Miguel Servet, Zaragoza, Spain; 36 Department of Anesthesiology, University Hospital of León, León, Spain; 37 Department of Anesthesiology, University Hospital 12 de Octubre, Madrid, Spain; 38 Department of Anesthesiology, University Hospital of Basurto, Bilbao, Spain; 39 Department of Anesthesiology, Ferrol University Hospital Complex, Ferrol, Spain; 40 Department of Anesthesiology, University Hospital Puerta del Hierro, Madrid, Spain; 41 Department of Anesthesiology, University Hospital of Burgos, Burgos, Spain; 42 Department of Anesthesiology, Central University Hospital of Asturias, Oviedo, Spain; 43 Department of Anesthesiology, University Hospital San Cecilio, Granada, Spain; 44 Unit of Research, Clinic University Hospital of Valladolid, Valladolid, Spain; 45 Department of Anesthesiology, University Hospital of Tajo, Aranjuez, Spain; 46 Department of Anesthesiology, Monforte de Lemos Hospital, Lugo, Spain; 47 Department of Anesthesiology, University Hospital Complex of Ourense, Ourense, Spain; 48 Department of Anesthesiology, Quirón Salud Valle del Henares Hospital, Torrejón de Ardoz, Spain; 49 Department of Anaesthesiology, University Hospital of Pontevedra, Pontevedra, Spain; 50 Department of Anesthesiology and Surgical-Trauma Intensive Care, Hospital Clinic Universitari de València, Valencia, Spain; 51 INCLIVA Biomedical Research Institute, Valencia, Spain; 52 Biomedical Engineering Group, University of Valladolid, Valladolid, Spain; 53 Biomedical Research Networking Center in Bioengineering, Biomaterials and Nanomedicine (CIBER-BBN), Carlos III Health Institute, Madrid, Spain; 54 Institute of Sanitary Research Galicia Sur IISGS, Galicia, Spain

**Keywords:** blood transfusion, DELPO, patient blood management, quality of health, Spain

## Abstract

**Background:**

Since their implementation in Spain, adherence of hospitals to Patient Blood Management (PBM) programs has been variable, potentially influencing transfusion practices. This study aimed to evaluate, in a real-world surgical setting, the frequency and appropriateness of red blood cell (RBC) transfusion.

**Methods:**

A prospective multicenter study in 43 Spanish hospitals including surgical patients. Transfusion appropriateness was evaluated using evidence-based criteria based on hemoglobin thresholds and clinical conditions such as cardiovascular disease, acute hemorrhage, or high comorbidity burden. Statistical analyses identified factors associated with transfusion practices.

**Results:**

The overall perioperative RBC transfusion rate was 9.7%, with the highest rates in cardiac (52.9%), vascular (17.9%), and orthopedic (12.3%) surgeries. RBC transfusion was associated with older patients with comorbidities, lower preoperative hemoglobin, higher ASA score and worse surgical meters and postoperative outcomes. Transfused patients showed significantly lower 60-day survival. Critically, 43% of transfusions were inappropriate, while transfusion omission (1.9%) may represent a clinical concern that warrants further investigation. Inappropriate transfusion was more frequent in older comorbidity patients according to Charlson Comorbidity Index in urgent surgery. In multivariable analysis, age was a factor associated with inappropriate transfusion, by cons, surgical blood loss was the main protective factor against inappropriate transfusion.

**Conclusion:**

As far as we know, this is the first Spanish multicenter study evaluating transfusion appropriateness in surgical scenario. Despite a lower overall transfusion rate than international figures, nearly half of transfusions were inappropriate and transfusion omission, also represents a real clinical concern. Implementation of decision-support tools and strengthened PBM protocols are needed to address factors associated with inappropriate transfusion, such as age, and to optimize patient safety and resource use.

## Introduction

Blood transfusion is one of the most frequent interventions in surgery, accounting for 24–44% of all red blood cell (RBC) units administered during hospitalization, and up to 50% in major surgery (1). Although it aims to correct tissue hypoxia, transfusion could carry several risks, including circulatory overload, immune reactions, viral transmission, alloimmunization, increased postoperative morbidity and mortality, and the potential for human error (2). Moreover, it is a limited and costly resource: in Spain, each unit costs €125–150 (3), in addition to medical and hospital-related expenses (4).

In cardiac surgery transfusion rates may reach 40–50%, whereas in non-cardiac surgery (including major abdominal one) they range from 10 to 40% (1–3, 5, 6). In orthopedic surgery, between one-quarter and one-third of patients with hip fracture receive transfusion, often outside recommended thresholds. High variability in transfusion practices reported in these studies is not fully explained by patient severity, suggesting unwarranted differences in clinical practice (1).

Patient blood management (PBM) has been defined as a patient-centered, systematic, evidence-based approach to improve patient outcomes by managing and preserving a patient’s own blood, while promoting patient safety and empowerment (7). With a structured, multimodal approach it is based on three pillars to (1) optimize the red-blood cell mass, (2) minimize blood loss and (3) properly manage anemia during the pre-, intra, and post-operative phase, aiming at optimizing blood use, reducing unnecessary transfusions, and improving clinical outcomes (8–12). Its implementation has been associated with lower transfusion rates, fewer infectious complications, and reduced 90-day mortality without increasing any kind of complications (13).

Despite this evidence, PBM adoption remains uneven, hindered by clinical inertia, poor interoperability of health information systems, and limited specialized training (14–16). These barriers contribute to inappropriate transfusion practices, both inappropriate transfusion and omission (14–16). Even with well-established guidelines (11), 18–57% of RBC transfusions may be unnecessary (14, 17).

In this regard, we aimed to assess, under real-world conditions, the frequency and appropriateness of RBC transfusion, as well as the impact of PBM programs in the surgical context in Spain.

## Materials and methods

### Study design

A prospective, multicenter, and observational study conducted in 43 Spanish hospitals between November 14 and 21, 2023 (18). The study protocol was approved by the Ethics Committee of Hospital Universitario de Pontevedra (code PI 2021/479) and by the local ethics committees of all participating centers. Written informed consent was obtained from all patients. The study is registered at ClinicalTrials.gov (NCT06127901) and the present manuscript adheres to the Strengthening the Reporting of Observational Studies in Epidemiology (STROBE) guidelines.

### Study population

We included patients aged ≥ 18 years undergoing urgent or elective surgery requiring hospital admission under any type of anesthesia. Consecutive inclusion was performed, and data was collected using a standardized electronic case report form on the REDCap platform.

For this analysis, only patients with complete data on preoperative hemoglobin, lowest hemoglobin value (nadir) recorded on the first postoperative day, nadir between postoperative days 2 and 7, post-transfusion hemoglobin, and number of RBC units administered were selected. Fresh frozen plasma (FFP) or platelet administration were also recorded. Cases with missing data for these variables were excluded.

Firstly, the cohort was divided into two groups: (a) patients who received at least one unit of RBCs throughout the admission (both intra- and postoperative moments), (b) patients who were not RBC transfused. This comparison allowed us to explore clinical and organizational factors associated with transfusion. Secondly, appropriateness of RBC transfusion was analyzed in transfused patients. This let us define the rates of inappropriate transfusion or omission, as well as the key factors for these reasons.

Patients received the necessary standard medical care for their surgical interventions at their respective hospitals, including anesthetic procedures and postoperative care. Preoperative, intraoperative, and postoperative variables for all patients were recorded.

### Clinical endpoints

The primary endpoint of the study was to define the rates of RBC transfusion (global rate and each hospital separately) and appropriateness as well as the factors associated with. Secondary endpoints included hospital meter analysis associated with transfusion.

### Variables

We included age, sex, relevant comorbidities including Charlson Comorbidity Index, ASA classification system, infection (defined as any documented infection during the index hospitalization that required antibiotic therapy), and hospital meters (60-day mortality, hospital and ICU stay). Surgical specialties were separately included into 12 types (digestive surgery, urological surgery, gynecological surgery, otorhinolaryngology surgery, maxillofacial surgery, neurosurgery, cardiac surgery, vascular surgery, plastic surgery, orthopedic surgery, ophthalmologic surgery and other surgery). We also collected transfusion-related parameters:

1. Preoperative hemoglobin level.
2. Lowest hemoglobin level in two moments: early postoperative (day 1) and late postoperative (days 2–7).
3. Type of surgery and urgency status (elective or urgent)
4. Use of vasoactive drugs during intraoperative and early postoperative period, quantification of surgical blood loss, intubation and surgery time.
5. Number of RBC concentrates and number of FFP or platelet pools. These data were collected reliably through the blood banks of each hospital (Hematology and Hemotherapy department).

### Criteria for transfusion appropriateness

The criteria for transfusion appropriateness were developed based on the Frankfurt Consensus Conference and AABB guidelines (11, 17, 19), with some modifications to reflect clinical complexity. While a hemoglobin threshold of < 7 g/dL (criterion A) is fully aligned with restrictive strategies, we included additional clinical conditions to capture real-world decision-making (criterion B). Specifically, a threshold of 7–8 g/dL was applied to patients with cardiovascular or cerebrovascular disease (consistent with weak recommendations for this population), and to those undergoing orthopedic, vascular, or cardiac surgery (a common extrapolation in high-risk procedures). Respiratory disease, peripheral oxygen saturation ≤ 91%, and a Charlson Comorbidity Index ≥ 5 were included based on the rationale that these factors may impair oxygen delivery or reflect limited physiological reserve, even though they are not explicitly endorsed in the cited guidelines. Acute hemorrhage (criterion C) was defined using a composite of blood loss and hemoglobin levels to approximate the dynamic nature of intraoperative bleeding. We acknowledge that these modifications may influence the classification of appropriateness and this is addressed in the limitations.

RBC transfusion was considered appropriate if at least one of the following criteria was met. Based on those criteria, inappropriate transfusion was defined as RBC transfusion in the absence of the above criteria, meanwhile omission was the absence of RBC transfusion meeting at least one transfusion criteria.

### Statistical analysis

Quantitative variables were summarized as median and interquartile range (IQR) after confirming non-normal distribution with the Kolmogorov–Smirnov test. Categorical variables were expressed as absolute frequencies and percentages. Group comparisons (transfused vs. non-transfused patients, and appropriate vs. non-appropriate transfusions) were performed using the Mann–Whitney U test for continuous variables and the Pearson χ^2^ test or Fisher’s exact test when required for categorical variables.

Univariate binary logistic regression was conducted to identify factors associated with RBC transfusion as well as to identify the critical factors associated with transfusion appropriateness. Variables with *p* < 0.10 in univariate analysis were considered for multivariable modeling. Before model inclusion, collinearity was assessed using Pearson’s correlation coefficient, excluding highly correlated variables (*r* > 0.80).

The multivariable logistic regression model was built using backward stepwise elimination based on the Wald criterion, with an exclusion threshold of *p* > 0.10. Results were expressed as adjusted odds ratios (aOR) with 95% confidence intervals (CI). 60-day survival analysis was performed using Kaplan–Meier curves and Cox analysis model, compared by log-rank test.

All statistical analyses were performed with R software (version 4.3.2) and SPSS (version 29). A *p*-value < 0.05 was considered statistically significant.

## Results

Our study included 2,442 patients and 237 received RBC transfusion at some point during hospitalization, showing a 9.7% perioperative transfusion rate. Transfusion rates across centers ranged between 0 and 26.1%, however, there were statistically significant differences across hospitals (*p* = 0.003), as well as the sample sizes were highly heterogeneous and limited in many cases. Those transfused patients were also divided into 90 patients (3.7%) transfused intraoperatively, 101 (4.1%) within the first 24 postoperative hours, and 131 (5.4%) between postoperative day 2 and hospital discharge. Some patients received RBC transfusion in two moments, including 17 patients in all 3 different moments. The median number of RBC concentrates transfused per patient was 2 (IQR: 1–2). In addition, 21 patients (0.86%) received platelet transfusion, 21 patients (0.86%) received FFP, and 8 patients received both platelet and FFP. All cases were accompanied by RBC transfusion.

Table 1 summarizes demographic and clinical characteristics of patients according to RBC transfusion. Transfused patients were significantly older (71 vs. 63 years; *p* < 0.001) and had a higher burden of comorbidities: hypertension (59.5% vs. 42.7%; *p* < 0.001), heart disease (26.2% vs. 9.6%; *p* < 0.001), peripheral vascular disease (13.9% vs. 5.5%; *p* < 0.001), chronic kidney disease (9.7% vs. 2.4%; *p* < 0.001), and cerebrovascular disease (9.3% vs. 5.6%; *p* = 0.024), as well as a higher Charlson Comorbidity Index (median 3 vs. 2; *p* < 0.001).

**TABLE 1:** Characteristic of transfused and non-transfused patients.

Characteristics	Transfusion (*N* = 237)	Non-transfusion (*N* = 2205)	*p*-value
Age, years	71 (14)	63 (24)	< 0.001
Sex, male	110 (46.4%)	1066 (48.3%)	0.542
Comorbidities			
Cerebrovascular disease	22 (9.3%)	124 (5.6%)	0.024
Hypertension	141 (59.5%)	941 (42.7%)	< 0.001
Heart disease	62 (26.2%)	211 (9.6%)	< 0.001
Peripheral vascular disease	33 (13.9%)	121 (5.5%)	< 0.001
Respiratory disease	20 (8.4%)	134 (6.1%)	0.155
Renal disease	23 (9.7%)	52 (2.4%)	< 0.001
Charlson Index	3 (2)	2 (2)	< 0.001
Preoperative parameters			
ASA classification system	3 (1)	2 (1)	< 0.001
Hemoglobin level, g/dL	11.8 (3.5)	14 (2.3)	< 0.001
Urgent surgery	34 (14.3%)	168 (7.6%)	< 0.001
Surgical specialties			
Digestive surgery	39 (16.5%)	553 (25.1%)	< 0.001
Urological surgery	29 (12.2%)	298 (13.5%)	
Gynecological surgery	11 (4.6%)	167 (7.6%)	
Otorhinolaryngology surgery	1 (0.4%)	129 (5.9%)	
Maxillofacial surgery	4 (1.7%)	61 (2.8%)	
Neurosurgery	7 (3%)	120 (5.4%)	
Cardiac surgery	36 (15.2%)	32 (1.5%)	
Vascular surgery	21 (8.9%)	96 (4.4%)	
Plastic surgery	7 (3%)	68 (3.1%)	
Orthopedic surgery	76 (32.1)	541 (24.5%)	
Ophthalmologic surgery	0 (0%)	8 (0.4%)	
Other surgery	6 (2.5%)	132 (6%)	
Intraoperative parameters			
Vasoactive agents	115 (50.2%)	336 (17.1%)	< 0.001
Surgical blood loss, ml	300 (600)	100 (170)	< 0.001
Mechanical ventilation time, min	252.5 (210)	120 (115)	< 0.001
Surgery time, min	180 (200)	95 (90)	< 0.001
Postoperative parameters			
Infection	87 (36.7%)	276 (12.5%)	< 0.001
Lowest hemoglobin level, g/dL	8.1 (1.6)	12.2 (2.2)	< 0.001
ICU stay, days	3 (7)	1 (1)	< 0.001
Hospital stay, days	12 (19)	2 (4)	< 0.001
60-day mortality	11 (4.6)	11 (0.5)	< 0.001

ASA, American Society of Anesthesiologists; ICU, Intensive Care Unit. Quantitative variables were described using median values and the interquartile range (IQR), while categorical variables were presented as total numbers (n) and percentages (%).

Preoperatively, transfused patients had lower hemoglobin levels (11.8 vs. 14 g/dL; *p* < 0.001), higher anesthetic risk according to ASA classification (3 vs. 2; *p* < 0.001), and a greater proportion of urgent interventions (14.3% vs. 7.6%; *p* < 0.001). Intraoperatively, they exhibited higher rates of vasoactive drug use (50.2% vs. 17.1%; *p* < 0.001), greater surgical blood loss (300 vs. 100 mL; *p* < 0.001), and longer mechanical ventilation times (252.5 vs. 120 min; *p* < 0.001) as well as longer surgical duration (180 vs. 95 min; *p* < 0.001).

Postoperatively, transfused patients showed a higher incidence of infection (36.7% vs. 12.5%; *p* < 0.001), lower hemoglobin levels (8.1 vs. 12.2 g/dL; *p* < 0.001), longer ICU (3 vs. 1 days; *p* < 0.001) and hospital stays (12 vs. 2 days; *p* < 0.001), and higher 60-day mortality (4.6% vs. 0.5%; *p* < 0.001).

The proportion of transfused patients varied widely according to surgical specialty (Table 2), being highest in cardiac surgery (52.9%), followed by vascular surgery (17.9%) and orthopedic surgery (12.3%), with rates below 10% in all other specialties.

**TABLE 2:** RBC transfusion rate according to the different surgical specialties.

Surgical specialties	Number of surgeries	RBC transfusion rate
Digestive surgery	592	39 (6.6%)
Urological surgery	327	29 (8.9%)
Gynecological surgery	178	11 (6.2%)
Otorhinolaryngology surgery	130	1 (0.8%)
Maxillofacial surgery	65	4 (6.2%)
Neurosurgery	127	7 (5.5%)
Cardiac surgery	68	36 (52.9%)
Vascular surgery	117	21 (17.9%)
Plastic surgery	75	7 (9.3%)
Orthopedic surgery	617	76 (12.3%)
Ophthalmologic surgery	8	0 (0.0%)
Other surgery	138	6 (4.3%)

RBC, Red blood cell

Supplementary Figure 1 shows that transfused patients generally had a more complex clinical profile, with higher baseline risk, more aggressive surgical procedures, and worse postoperative outcomes. Univariate logistic regression identified the following as the strongest predictors of RBC transfusion: cardiac surgery (OR = 7.42; *p* < 0.001), infection (OR = 3.68; *p* = 0.001), renal disease (OR = 3.37; *p* < 0.001), vasoactive agents (OR = 3.24; *p* = 0.001), heart disease (OR = 2.48; *p* < 0.001), urgent surgery (OR = 2.46; *p* = 0.001), peripheral vascular disease (OR = 1.91; *p* < 0.001), and ASA classification system (OR = 1.87; *p* = 0.001), and age (OR = 1.04; *p* < 0.001). Moreover, RBC transfusion was associated with higher 60-day mortality risk (OR = 4.87; *p* < 0.001).

The Multivariable Logistic Regression showed eight independent predictors of RBC transfusion (Supplementary Table 1). Lower preoperative hemoglobin was the strongest modifiable risk factor (aOR = 0.603 per g/dL increase). Other significant predictors included vasoactive agents (aOR = 2.005), infection (aOR = 2.185), urgent surgery, greater surgical blood loss, longer surgery time, specific surgery types, and older age (aOR = 1.025 per year). Sex, ASA score, and Charlson Index were not retained in the final model.

**FIGURE 1:**
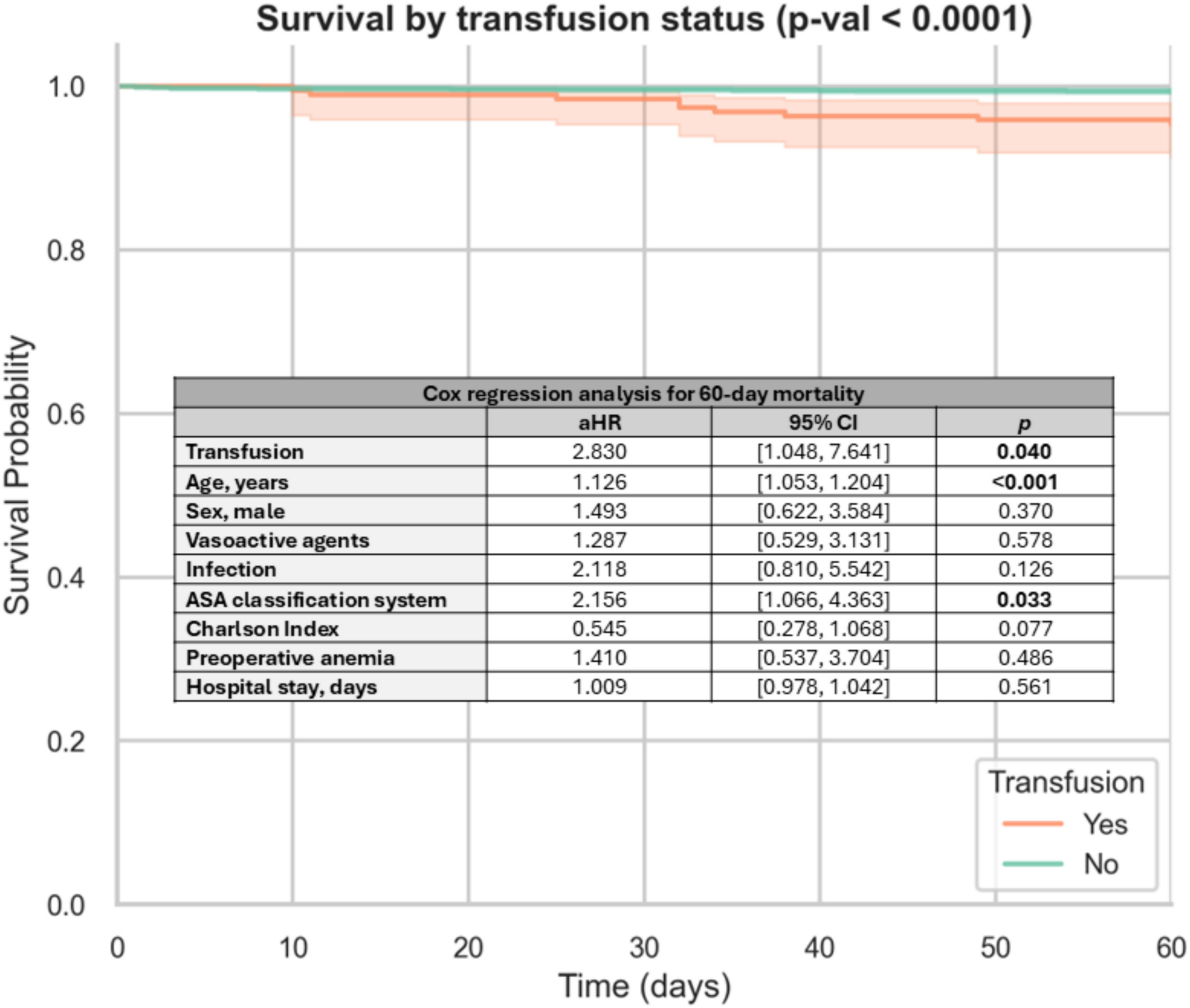
Kaplan Meier survival curves and Cox regression analysis for 60-day mortality according to RBC transfusion. aHR, adjusted hazard ratio; CI, confidence interval; ASA, American Society of Anesthesiologists.

The 60-day Kaplan–Meier survival curve (Figure 1) demonstrated lower cumulative survival in transfused patients compared with non-transfused patients (95.4% vs. 99.5%; log-rank: *p* < 0.001). The Cox Regression (60-day Mortality) demonstrated how RBC transfusion was independently associated with increased mortality risk (aHR = 2.830, *p* = 0.040), as well as age (aHR = 1.126) and higher ASA (aHR = 2.156). By cons, sex, infection, and hospital stay were not retained in the final model (Figure 1).

The appropriateness of RBC transfusion was explained in Table 3. Among the 237 transfused patients, 135 (57%) met the established clinical criteria for RBC transfusion (appropriate transfusion), whereas 102 (43%) were transfused without criteria (inappropriate transfusion). Among the 2,205 non-transfused patients, 41 (1.9%) met transfusion criteria but did not receive it (omission). Overall, 176 patients met transfusion criteria: 76.7% were transfused appropriately, while 23.3% were not transfused due to omission.

**TABLE 3:** Assessment of compliance with established criteria for transfusion of RBC concentrates.

*p* < 0.001		Transfusion of RBC concentrate	
		Yes (*N* = 237)	No (*N* = 2205)
Transfusion criteria	Yes (*N* = 176)	135 (57%)	41 (1.9%)
	No (*N* = 2266)	102 (43%)	2164 (98.1%)

RBC, Red blood cell.

(a) Meet transfusion criteria (*N* = 176): patients who meet transfusion criteria were divided between transfused—–Appropriate transfusion—–(*N* = 135) and non-transfused ones – transfusion omission—(*N* = 41). Appropriate transfusion showed lower preoperative hemoglobin level (11.9 g/dl vs. 13.2 g/dl; *p* < 0.001) as well as higher rate of preoperative anemia (55.6% vs. 31.7%; *p* = 0.007) compared to transfusion omissions. Moreover, appropriate transfusion showed longer hospital (15 days vs. 7 days; *p* < 0.001) and UCI stay (3 vs. 1; *p* = 0.001) and higher rate of infection (36.3% vs. 14.6%; *p* = 0.008).

(b) Transfused patients (*N* = 237): Table 4 showed the characteristics of RBC transfused patients according to the compliance with appropriate transfusion. Appropriate transfusion according to criteria was evidenced in younger patients (69 vs. 74 years; *p* = 0.004) with lower Charlson Comorbidity Index scores (3 vs. 4; *p* = 0.028), highlighting a lower prevalence of hypertension (52.6% vs. 68.6%; *p* = 0.013). They received more RBC units (2 vs. 1; *p* < 0.001), experienced greater intraoperative blood loss (500 mL vs. 275 mL; *p* < 0.001), and more frequently required vasoactive drugs (57.8% vs. 40.2%; *p* = 0.007). In addition, their surgeries were longer (209 vs. 150 min; *p* = 0.006), they were more often transfused beyond the first 24 postoperative hours (61.7% vs. 43.2%; *p* = 0.013) and had lower lowest postoperative hemoglobin values (7.6 vs. 8.8 g/dL; *p* < 0.001). In contrast, inappropriate transfusion was more frequent in older comorbidity patients (mainly hypertension) in urgent surgery (19.6% vs. 10.4%; *p* = 0.045). No significant differences were observed between groups in 60-day mortality, infection rates, ICU stay or hospital length of stay.

**TABLE 4:** Characteristic of RBC transfused patients according to the compliance with appropriate transfusion (see in methods section).

Characteristics	Appropriate transfusion (*N* = 135)	Non-Appropriate transfusion (*N* = 102)	*p*- value
Age, years	69 (19)	74 (18)	0.004
Sex, male	62 (45.9%)	68 (67.1%)	0.863
RBC transfusion parameters			
Number of RBC concentrates	2 (3)	1 (2)	< 0.001
RBC Intraoperative	52 (38.5%)	38 (37.3%)	0.843
RBC first 24h	63 (46.7%)	38 (33.3%)	0.144
RBC 2° day to discharge	84 (61.7%)	44 (43.2%)	0.013
Comorbidities			
Cerebrovascular disease	14 (10.4%)	8 (7.8%)	0.507
Hypertension	71 (52.6%)	70 (68.6%)	0.013
Heart disease	33 (24.4%)	29 (28.4%)	0.489
Peripheral vascular disease	16 (11.9%)	17 (16.7%)	0.289
Respiratory disease	10 (7.4%)	10 (9.8%)	0.511
Renal disease	13 (9.6%)	10 (9.8%)	0.964
Charlson index	3 (2)	4 (1)	0.028
Preoperative parameters			
ASA classification system	3 (1)	3 (1)	0.049
Hemoglobin level, g/dL	12 (3.5)	11.6 (3.5)	0.545
Urgent surgery	14 (10.4%)	20 (19.6%)	0.045
Surgical specialties			
Digestive surgery	22 (16.3%)	17 (16.7%)	0.447
Urological surgery	11 (8.1%)	18 (17.6%)	
Gynecological surgery	8 (5.9%)	3 (2.9%)	
Otorhinolaryngology surgery	0 (0%)	1 (1%)	
Maxillofacial surgery	4 (3%)	0 (0%)	
Neurosurgery	3 (2.2%)	4 (3.9%)	
Cardiac surgery	23 (17%)	13 (12.7%)	
Vascular surgery	12 (8.9%)	9 (8.8%)	
Plastic surgery	5 (3.7%)	2 (2%)	
Orthopedic surgery	42 (31.1)	34 (33.3%)	
Ophthalmologic surgery	0 (0%)	0 (0%)	
Other surgery	5 (3.7%)	1 (1%)	
Intraoperative parameters			
Vasoactive agents	78 (57.8%)	41 (40.2%)	0.007
Surgical blood loss, ml	500 (700)	275 (300)	< 0.001
Mechanical ventilation time, min	276.5 (208)	212.5 (221)	0.077
Surgery time, min	209 (200)	150 (150)	0.006
Postoperative parameters			
Infection	49 (36.3%)	38 (37.3%)	0.880
Lowest hemoglobin level, g/dL	7.6 (0.8)	8.8 (1.3)	< 0.001
ICU stay, days	3 (8)	2 (6)	0.083
Hospital stay, days	15 (21)	10 (15)	0.120
60-day mortality	6 (4.4%)	5 (4.9%)	0.868

RBC, Red blood cell; ASA, American Society of Anesthesiologists; ICU, Intensive Care Unit. Quantitative variables were described using median values and the interquartile range (IQR), while categorical variables were presented as total numbers (n) and percentages (%).

Supplementary Figure 2 shows the Pearson correlation matrix for pre-, intra-, and postoperative variables significant in univariate analysis, aiming to identify potential collinearity. A very strong correlation was observed between age and Charlson Comorbidity Index (*r* = 0.84), as well as between mechanical ventilation time and surgical duration (*r* = 0.82).

A final multivariable model (Supplementary Table 2) was developed using backward stepwise elimination with Wald criterion. This model identified age as the main non-clinical risk factor associated with inappropriate transfusion, with a 3% increase in probability per additional year (aOR = 1.030; 95%CI: 1.008–1.053; *p* < 0.008), while quantified intraoperative blood loss was associated with a lower risk of inappropriate transfusion (aOR = 0.998; 95%CI: 0.997–0.999; *p* < 0.001).

## Discussion

This multicenter study, analyzing transfusion practices in surgical patients requiring hospitalization across 43 hospitals distributed throughout Spain, presents three main findings: (i) the overall RBC transfusion rate was 9.7%; (ii) the rate of inappropriate transfusions was 43%, while 1.9% of patients who met the criteria were not transfused (omission); and (iii) surgical blood loss was identified as an expected clinical factor associated with transfusion, whereas age was a factor associated with inappropriate transfusion.

In the literature, transfusion rates in major surgery range between 15 and 30%, varying according to the type of procedure, patient condition, and transfusion policies (6, 14, 20). In a German cohort of 1.2 million patients, Meybohm et al. reported rates of 10.5% before and 9.4% after implementing a PBM program (13). In non-cardiac surgery, Verret et al. reported ranges of 10–40% (21), whereas in orthopedic or cardiac surgery, rates can exceed 50% in older patients or those with high comorbidity (22). In Spain, the ARCA-1 study, focused on major oncologic surgery, reported a 21.1% rate (23). Our overall rate (9.7%) is low, comparable to that of Meybohm et al. (13), and at the lower limit of the range described by Verret et al. (21). This likely could reflect the widespread implementation in Spain—over the past decade—of PBM strategies (24) and ERAS protocols, which include preoperative hemoglobin optimization, strict hemostatic control, minimally invasive techniques, and tranexamic acid use (14, 21, 25).

This likely could reflect the widespread implementation in Spain—over the past decade—of PBM strategies which include preoperative hemoglobin optimization, strict hemostatic control, minimally invasive techniques, and tranexamic acid (24) and the application of ERAS protocols in most hospitals and surgeries.

By specialty, we found lower transfusion rates than those reported in the literature for digestive, otolaryngologic, and maxillofacial surgery, and similar figures in vascular and orthopedic surgery. Regarding timing, transfusion was most common between postoperative day 2 and hospital discharge (5.4%), followed by the first postoperative 24 h (4.1%) and the intraoperative period (3.7%). This pattern is consistent with recent series (26–28) and reflects more restrictive intraoperative policies, with indications deferred until anemia progression is confirmed, combined with closer postoperative monitoring to detect occult bleeding (25).

The clinical profile of our transfused patients aligns with previous reports: older age, higher comorbidity burden, worse ASA class, greater blood loss, longer hospital stays, and higher mortality (2, 22, 27). These findings reinforce the adoption of individualized PBM strategies.

Patient blood management (PBM) has been defined as a patient-centered, systematic, evidence-based approach to improve patient outcomes by managing and preserving a patient’s own blood and is now considered the standard of care in transfusion medicine, as endorsed by the World Health Organization (29). However, inappropriate transfusion, particularly the administration of blood products without justification—remains common. Jadwin et al. (17) estimated that 45% of transfusions could have been avoided and that nearly half of all administered components were unnecessary, with an impact on hospital length of stay. In hip arthroplasty in patients aged ≥ 65 years, Kim et al. (14) showed that transfusion appropriateness increased from 54.0 to 94.7% after PBM implementation. In our cohort, 43% of transfused patients did not meet clinical criteria, suggesting a relevant degree of overuse, similar to that reported in critical care settings where transfusion occurs above recommended hemoglobin thresholds (30).

At the other extreme, transfusion omission—failure to administer blood despite its indication—has received less attention. Tan et al. (31) observed that delayed transfusion after radical cystectomy was associated with increased 90-day mortality. In our study, 1.9% of patients were not transfused despite meeting criteria, and overall, one in four patients with an indication did not receive transfusion, highlighting omission as a real clinical problem requiring targeted interventions.

Multivariable analysis identified age as the main predictor of inappropriate transfusion, with a 3% increase in probability per additional year, consistent with previous studies (2, 17). This finding may reflect that age serves as a marker for unmeasured factors such as frailty, cognitive impairment, or limited physiological reserve, which may influence clinical judgment (32). It is also possible that some transfusions classified as inappropriate in older patients reflect cautious clinical decision-making rather than bias. Our data does not allow us to distinguish between these possibilities. Conversely, quantified intraoperative blood loss emerged as a protective factor, consistent with the notion that objective estimation of blood loss supports more appropriate decision-making (13, 17, 23).

Both deviations—inappropriate transfusion and omission—carry risks: from circulatory overload (24, 27) to tissue hypoxia and poorer postoperative outcomes (24, 25). Their coexistence points to systemic weaknesses that should be addressed through decision-support tools, audits, indicators, and continuous training (21, 25, 33). PBM programs have proven effective in reducing unnecessary transfusions without compromising patient safety (29, 33).

Our findings are directly applicable to surgical settings and may serve as a reference for healthcare systems seeking to optimize blood product utilization. The observation that age was associated with inappropriate transfusion raises the question of whether current restrictive thresholds (Hb 7–8 g/dL) are appropriately applied to older patients with complex comorbidities. Our results could move to include patient-specific factors such as life expectancy or age-related comorbidities for the suitable decision for transfusion in elderly patients.

The main strengths of this study lie in its multicenter design, including 43 hospitals across different levels of care and geographical regions in Spain, which provides a broad and realistic picture of current transfusion practices. The large sample size allowed for robust analyses, including multivariable modeling to control confounding factors. Importantly, the study simultaneously assessed both transfusion frequency and appropriateness in a wide range of surgical specialties, something rarely addressed in the literature. The use of standardized criteria for transfusion evaluation, together with adherence to STROBE recommendations, enhances the methodological rigor. Overall, these features confer high external validity to our findings and make them a valuable reference for healthcare systems aiming to optimize blood utilization within PBM programs.

This study also has several limitations. First, its *post hoc* and observational design precludes establishing causal relationships between transfusion practices and outcomes. Second, the assessment of appropriateness was based on standardized international criteria, but clinical judgment in real-world scenarios may incorporate additional factors not captured in the database, which could have led to misclassification. Third, the definition of acute hemorrhage was based on surgical blood loss and hemoglobin thresholds, but we did not capture dynamic intraoperative factors such as hemodynamic instability or rapid hemoglobin decline, which may have led to misclassification of some transfusions as inappropriate when they were clinically justified. Fourth, the assessment of appropriateness was based on standardized international criteria with some modifications to capture real-world clinical complexity; however, these modifications may have led to misclassification of some transfusions. Fifth, using a single postoperative nadir hemoglobin value to define omission may have overestimated the rate of omitted transfusions by including transient, clinically insignificant low Hb values that did not warrant intervention. Finally, these results reflect the Spanish healthcare setting, where PBM programs are widely implemented; therefore, extrapolation to other health systems should be made with caution.

## Conclusion

This study shows that transfusion rates in Spain are lower than those reported in the international literature, likely reflecting the sustained impact of PBM programs implemented over the past decade. Nevertheless, important areas for improvement remain due to inappropriate RBC transfusion still affecting nearly half of all transfusions. Reducing the influence of factors—such as the association between age and inappropriate transfusion—and reinforcing the objective assessment of surgical blood loss should be a priority to optimize patient safety, improve clinical outcomes, and ensure the efficient use of healthcare.

## Data Availability

The raw data supporting the conclusions of this article will be made available by the authors, without undue reservation.
