# Supplementary material for: High inappropriate red blood cell transfusion rate despite low overall use: a real-world multicenter study in 43 Spanish hospitals
Source: Front Med (Lausanne). 2026 May 14;13:1803092. doi: 10.3389/fmed.2026.1803092 (PMC13216454; doi:10.3389/fmed.2026.1803092)
Supplement: Supplementary file 1 [file Supplementary_file_1.DOCX]

**Supplementary material**

**Supplementary Figure 1:** Univariate analysis showing the variables associated with transfusion.

**
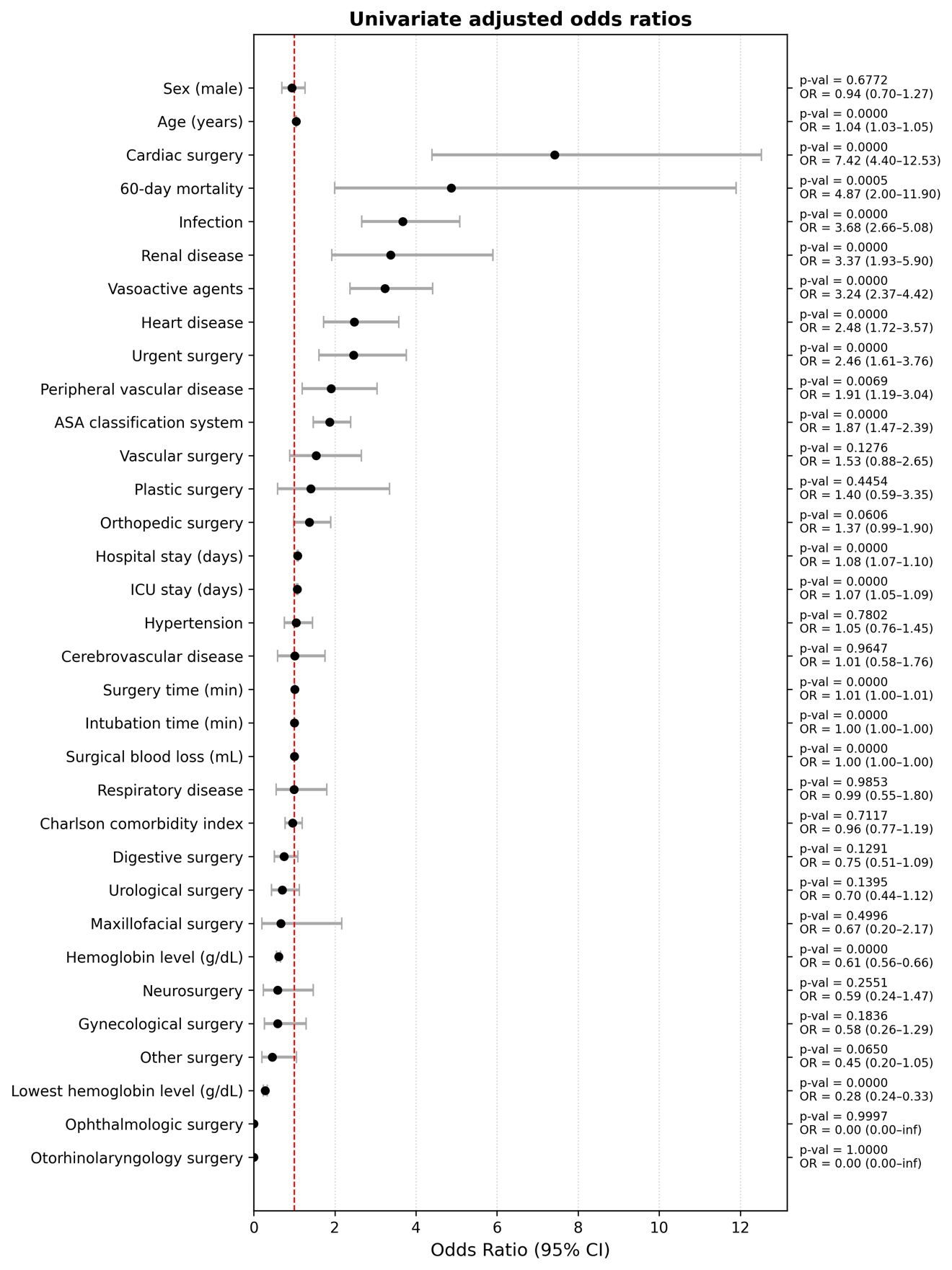
**

**Supplementary Table 1:** Multivariable Logistic Regression Model showing the determinants of RBC Transfusions.

|  | **aOR** | **95% CI** | ***p*** |
| --- | --- | --- | --- |
| **Age, years** | 1.025 | [1.003, 1.047] | **0.024** |
| **Infection** | 2.185 | [1.511, 3.160] | **<0.001** |
| **Urgent surgery** | 1.851 | [1.089, 3.148] | **0.023** |
| **Preoperative hemoglobin level, g/dL** | 0.603 | [0.551, 0.659] | **<0.001** |
| **Surgery type** | 1.075 | [1.028, 1.124] | **0.001** |
| **Surgical blood loss, ml** | 1.002 | [1.001, 1.002] | **<0.001** |
| **Vasoactive agents** | 2.005 | [1.401, 2.868] | **<0.001** |
| **Surgery time, min** | 1.005 | [1.004, 1.007] | **<0.001** |

Variables not retained in the final model: Sex (p=0.398), ASA score (p=0.273), Charlson Comorbidity Index (p=0.476). aOR, adjusted odds ratio; CI, confidence interval.

**Supplementary Figure 2:** Pearson’s correlation analysis


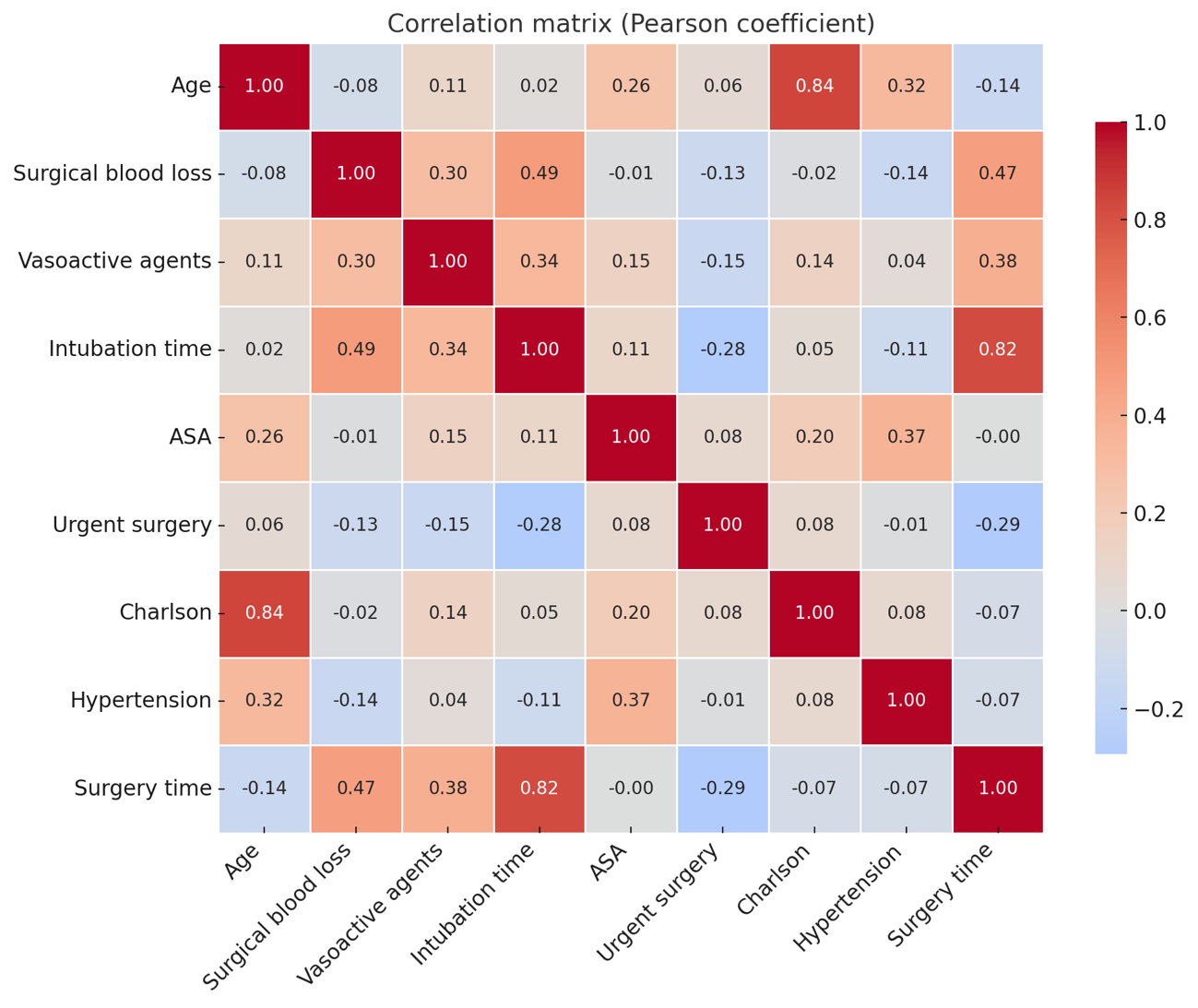


**Supplementary Table 2:** Multivariable Logistic Regression Model showing the determinants of Non-Criteria RBC Transfusions.

|  | **aOR** | **95% CI** | ***p*** |
| --- | --- | --- | --- |
| **Age (per year)** | 1.030 | [1.008, 1.053] | **0.008** |
| **Surgical blood loss (per ml)** | 0.998 | [0.997, 0.999] | **<0.001** |

RBC, Red blood cell; aOR, adjusted odds ratio; CI, confidence interval. Model developed using backward stepwise elimination with Wald criterion (removal threshold p>0.10).
